# The Effect of Phytochemicals and Food Bioactive Compounds on Diabetes

**DOI:** 10.3390/ijms23147765

**Published:** 2022-07-14

**Authors:** Kazumi Yagasaki, Christo J. F. Muller

**Affiliations:** 1 Division of Applied Biological Chemistry, Institute of Agriculture, Tokyo University of Agriculture and Technology, Tokyo 183-8509, Japan; 2 Biomedical Research and Innovation Platform (BRIP), South African Medical Research Council (MRC), Tygerberg, Cape Town 7505, South Africa; 3 Centre for Cardio-Metabolic Research in Africa, Division of Medical Physiology, Faculty of Medicine and Health Sciences, Stellenbosch University, Tygerberg, Cape Town 7505, South Africa; 4 Department of Biochemistry and Microbiology, University of Zululand, KwaDlangezwa, Empangeni 3886, South Africa

There are three main types of diabetes, namely, type 1 diabetes, type 2 diabetes (T2D), and diabetes in pregnancy (gestational diabetes). Of these, T2D accounts for approximately 90% of all diabetes cases [1], and the rising trend can be attributed to aging, urbanization, unhealthy lifestyle and the obesogenic environment [1]. Diabetes, especially the global prevalence of T2D, is increasing, as reported in the Diabetes Atlas, 9th and 10th editions in 2019 and 2021, respectively. In 2019, it was estimated that 463 million people had diabetes [1]. In 2021, the global diabetes prevalence in 20–79-year-olds was estimated to be 537 million people, and this number is projected to rise to 783 million in 2045 [2].

Screening antidiabetic components from edible natural products and elucidating their modes of action are considered to be an effective practice in terms of establishing their safety and validating their health benefits, which are reflected in their long histories of use of medicines. This Special Issue presents recent studies on the preventive and/or alleviating effects of various biofactors against diabetes, especially T2D, and diabetes-related disorders, as well as their modes of actions at molecular, cellular, tissue and/or whole-body levels. In this issue, biofactors include both non-nutrients and nutrients.

This Special Issue, entitled “The Effect of Phytochemicals and Food Bioactive Compounds on Diabetes”, was launched in 2021 with the publication of nine papers, including five review articles and four original articles describing the regulation of high blood glucose levels by biofactors in the diabetic state. The management of blood glucose levels and other metabolic imbalances that develop in T2D by these biofactors is of major importance and is discussed in detail in this Special Issue.

Insulin is central to the regulation of blood glucose levels. Rosenzweig and Sampson reviewed phytochemicals found to improve insulin sensitivity by the activation of the insulin signaling cascade and described the active constituents and their mechanisms of action by focusing on medicinal plants whose insulin-sensitizing properties are supported by both in vitro and in vivo data, such as penta-galloyl-glucose (PGG) [3]. Hira et al. reviewed the relationship between glucagon-like peptide-1 (GLP-1), a gastrointestinal hormone released from enteroendocrine L cells in response to meal ingestion and food factors, especially dietary peptides and flavonoids. Increasing endogenous GLP-1 secretion by using drugs or food factors could be a novel and promising strategy for preventing or improving glucose intolerance. Thus, in the review, the authors focused on the effects of dietary factors on GLP-1 stimulation [4]. Further glucose-induced insulin secretion (GSIS) is augmented by GLP-1 in response to ingesting nutrients. The secretion of insulin and GLP-1 is mediated by the binding of nutrients to G protein-coupled receptors (GPCRs) expressed by pancreatic β-cells and enteroendocrine L cells, respectively. Therefore, insulin secretagogues and incretin mimetics currently serve as antidiabetic treatments. Drzazga et al. studied and demonstrated the potency of synthetic isoprenoid derivatives of lysophosphatidylcholines (LPCs) to stimulate GSIS and GLP-1 release [5].

Szałabska-Rąpała et al. reviewed the antidiabetic effects of magnolol, a lignan from Magnolia bark, on diabetes, including its complications and comorbidities. Magnolol is thus a plant-derived, natural compound that shows promising effects in the prevention and treatment of T2D and its complications. The authors suggest magnolol may be a good supportive compound complementing conventional therapeutic methods in counteracting diabetic negative effects on the organism [6]. The herbal tea, rooibos, produced from *Aspalathus linearis*, is the only dietary source to date of aspalathin, a flavonoid and *C*-glucosyl dihydrochalcone. Muller and co-authors reviewed aspalathin and related compounds from various aspects of phytochemistry and bioactivty, then described recent studies on emerging therapeutic targets related to the preventive and alleviating effects and mechanisms of aspalathin and related compounds against T2D and diabetes-related disorders [7]. Kalai et al. reviewed the impact of isorhamnetin, an *O*-methylated flavonol, on diabetes-related disorders by decreasing glucose levels, ameliorating excessive oxidative stress, alleviating inflammation, modulating the lipid metabolism and adipocyte differentiation as reported in both in vitro and in vivo studies. In addition, these authors included a post hoc whole-genome transcriptome analysis of biological activities of isorhamnetin using a stem cell-based tool [8]. Watanabe et al. examined the effect of baccharin, a major component of Brazilian green propolis, on adipocyte differentiation. The treatment of mouse 3T3-L1 preadipocytes with baccharin resulted in increased lipid accumulation, cellular triglyceride levels, glycerol-3-phosphate dehydrogenase activity, and glucose uptake. In diabetic ob/ob mice, the intraperitoneal administration of baccharin significantly improved blood glucose levels. These results suggest that baccharin has a hypoglycemic effect on glucose metabolic disorders, such as type 2 diabetes mellitus [9]. Capsaicin in chili peppers has potential benefits for the reduction in glucose metabolism disorders and acts through the activation of transient receptor potential cation channel subfamily V member. Zinc, a micro nutrient, is essential for normal body functions, and the disruption of the zinc homeostasis is strongly associated with the pathogenesis of metabolic disorders. Ferdowsi and co-workers assessed capsaicin- and zinc-induced activation signaling molecules, and they found that capsaicin and zinc mediate glucose uptake in skeletal muscle cells occurs through the activation of calcium signaling [10].

Kim et al. [11] demonstrated that a protamex hydrolysate fraction (MLPH) from Meretrix lusoria (a saltwater clam), reduced body weight, hepatic steatosis and epididymal fat in obese ob/ob mice. Mechanistically, MLPH modulated the expression of key genes that regulate lipid and glucose metabolism and improved hepatic antioxidant status and glucose tolerance, suggesting that MLPH has anti-obesity and anti-hyperglycemic potential.

This Special Issue presents a selection of papers that focus on various bioactive factors and compounds that show promise in alleviating diabetes and related disorders. Scientific support is provided for their beneficial health effects, underlying mechanisms of action and therapeutic potential.

## References

[1] Saeedi P., Petersohn I., Salpea P., Malanda B., Karuranga S., Unwin N., Colagiuri S., Guariguata L., Motala A.A., Ogurtsova K. (2019). Global and regional diabetes prevalence estimates for 2019 and projections for 2030 and 2045: Results from the International Diabetes Federation Diabetes Atlas, 9th edition. Diabetes Res. Clin. Pract..

[2] Sun H., Saeedi P., Karuranga S., Pinkepank M., Ogurtsova K., Duncan B.B., Stein C., Basit A., Chan J.C.N., Mbanya J.C. (2022). IDF Diabetes Atlas: Global, regional and country-level diabetes prevalence estimates for 2021 and projections for 2045. Diabetes Res. Clin. Pract..

[3] Rosenzweig T., Sampson S.R. (2021). Activation of Insulin Signaling by Botanical Products. Int. J. Mol. Sci..

[4] Hira T., Trakooncharoenvit A., Taguchi H., Hara H. (2021). Improvement of Glucose Tolerance by Food Factors Having Glucagon-Like Peptide-1 Releasing Activity. Int. J. Mol. Sci..

[5] Drzazga A., Kamińska D., Gliszczyńska A., Gendaszewska-Darmach E. (2021). Isoprenoid Derivatives of Lysophosphatidylcholines Enhance Insulin and GLP-1 Secretion through Lipid-Binding GPCRs. Int. J. Mol. Sci..

[6] Szałabska-Rąpała K., Borymska W., Kaczmarczyk-Sedlak I. (2021). Effectiveness of Magnolol, a Lignan from Magnolia Bark, in Diabetes, Its Complications and Comorbidities—A Review. Int. J. Mol. Sci..

[7] Muller C.J.F., Joubert E., Chellan N., Miura Y., Yagasaki K. (2022). New Insights into the Efficacy of Aspalathin and Other Related Phytochemicals in Type 2 Diabetes—A Review. Int. J. Mol. Sci..

[8] Kalai F.Z., Boulaaba M., Ferdousi F., Isoda H. (2022). Effects of Isorhamnetin on Diabetes and Its Associated Complications: A Review of In Vitro and In Vivo Studies and a Post Hoc Transcriptome Analysis of Involved Molecular Pathways. Int. J. Mol. Sci..

[9] Watanabe A., de Almeida M.O., Deguchi Y., Kozuka R., Arruda C., Berretta A.A., Bastos J.K., Woo J.-T., Yonezawa T. (2021). Effects of Baccharin Isolated from Brazilian Green Propolis on Adipocyte Differentiation and Hyperglycemia in ob/ob Diabetic Mice. Int. J. Mol. Sci..

[10] Ferdowsi P.V., Ahuja K.D.K., Beckett J.M., Myers S. (2022). Capsaicin and Zinc Promote Glucose Uptake in C2C12 Skeletal Muscle Cells through a Common Calcium Signalling Pathway. Int. J. Mol. Sci..

[11] Kim M.J., Chilakala R., Jo H.G., Lee S.J., Lee D.S., Cheong S.H. (2022). Anti-obesity and Anti-hyperglycemic Effects of *Meretrix lusoria* Protamex Hydrolysate in *ob/ob* Mice. Int. J. Mol. Sci..

